# Practice Variation in the Management of First Trimester Miscarriage in The Netherlands: A Nationwide Survey

**DOI:** 10.1155/2014/387860

**Published:** 2014-11-04

**Authors:** Marianne A. C. Verschoor, Marike Lemmers, Malu Z. Wekker, Judith A. F. Huirne, Mariëtte Goddijn, Ben Willem J. Mol, Willem M. Ankum

**Affiliations:** ^1^Department of Obstetrics and Gynecology, Academic Medical Center, P.O. Box 22770, 1100 DE Amsterdam, The Netherlands; ^2^Department of Obstetrics and Gynaecology, Flevo Hospital, P.O. Box 3005, 1300 EG Almere, The Netherlands; ^3^Department of Obstetrics and Gynaecology, VUmc, P.O. Box 7057, 1007 MB Amsterdam, The Netherlands; ^4^The Robinson Institute, School of Paediatrics and Reproductive Health, University of Adelaide, 55 King William Road, North Adelaide, Adelaide, SA 5006, Australia

## Abstract

*Objectives*. To survey practice variation in the management of first trimester miscarriage in The Netherlands. *Methods*. We sent an online questionnaire to gynecologists in eight academic, 37 nonacademic teaching, and 47 nonteaching hospitals. Main outcome measures were availability of a local protocol; estimated number of patients treated with curettage, misoprostol, or expectant management; misoprostol regimen; and estimated number of curettages performed after initial misoprostol treatment. Outcomes were compared to the results of a previous nationwide survey. *Results*. The response rate was 100%. A miscarriage protocol was present in all academic hospitals, 68% of nonacademic teaching hospitals, and 38% of nonteaching hospitals (*P* = 0.008). Misoprostol was first-choice treatment for 41% of patients in academic hospitals versus 34% and 27% in teaching-and nonteaching hospitals (*P* = 0.045). There were 23 different misoprostol regimens. Curettage was first-choice treatment in 29% of patients in academic hospitals versus 46% and 50% in nonacademic teaching or nonteaching hospitals (*P* = 0.007). In 30% of patients, initial misoprostol treatment was followed by curettage. *Conclusions*. Although the percentage of gynaecologists who are aware of the availability of misoprostol for miscarriage treatment has doubled to almost 100% since 2005, practice variation is still large. This practice variation underlines the need for a national guideline.

## 1. Introduction

First trimester miscarriage is a frequent complication of pregnancy which occurs in 10–15% of pregnant women. In The Netherlands, this results in 18,000 to 27,000 miscarriages each year [1]. Because of the increased use of first trimester ultrasounds, ever more often the diagnosis nonvital pregnancy is made before symptoms occur [2]. In the past, Dutch women diagnosed with nonvital pregnancy either were managed expectantly, with complete expulsion of the pregnancy known to occur within two weeks in 37% of women, or were offered curettage [3]. However, in recent years, medical treatment with misoprostol has been shown to be an easy-to-use and inexpensive treatment option, which is well received by patients wishing to avoid surgical treatment. Treatment with misoprostol is effective in 53–99% of women with miscarriages, depending on dosage, route of administration, and duration of follow-up [4–8]. There are no significant differences between the use of misoprostol versus curettage on the rate of pelvic infections or ongoing subsequent pregnancies [9, 10]. Despite the high incidence of miscarriages and the developments in miscarriage treatment, a guideline from the Dutch Society of Obstetrics and Gynecology (NVOG) is still lacking. A national survey in 2005 among gynecologists in 92 hospitals that focused mainly on induced abortion but also studied spontaneous miscarriage showed that about 50% of Dutch gynecologists offered misoprostol as treatment option for first trimester miscarriages. However, there was a large practice variation with 27 different regimens being used for treatment of nonvital pregnancies [11]. Misoprostol could be a good alternative for curettage as first-choice treatment, but, to increase its effectiveness nationwide, all hospitals should offer the same clinically proven treatment regimens. In the present study, we investigated the current practice in miscarriage treatment in Dutch hospitals. Our aim was to survey the practice variation and especially to monitor the implementation of misoprostol, compared to the earlier survey performed in 2005.

## 2. Methods

We developed an online questionnaire which was sent by e-mail to practicing obstetricians and gynecologists in all Dutch hospitals, that is, eight academic, 37 nonacademic teaching, and 47 nonacademic nonteaching hospitals. The first mail was sent in December 2012, and reminders were sent monthly to nonresponders up until April 2013.

The questionnaire inquired about the estimated number of miscarriages per year, available treatment options, and the presence of a local protocol for managing miscarriages. Furthermore, we inquired how patients were informed about various treatment options, for example, by written information and/or by online information from the hospital's website.

Whenever medical treatment was offered, we asked for the dosages used, the route of administration, and the possible additional use of the antiprogesterone mifepristone. We also asked what percentage of patients underwent curettage after misoprostol use and what the main reason was for this intervention.

Analysis was performed per type of hospital: results of academic hospitals, nonacademic teaching hospitals, and nonteaching hospitals were compared. If estimated patient numbers provided by multiple respondents from the same hospital differed, we used the mean number in the analysis; incomplete and missing data were excluded from analysis.

Whenever possible, we compared the outcomes of the present questionnaire to the earlier nationwide survey, performed in 2005 and published in 2010. This previous study focussed mainly on induced abortion but also provided information on treatment of spontaneous miscarriage.

Chi-square tests and Fisher's exact test were used to compare categorical variables. To analyse differences in the use of misoprostol as first-choice treatment the Mann-Whitney *U* test was applied. A *P* value <0.05 was considered significant. All statistical analyses were done using the statistical package for social sciences (SPSS, IBM, Chicago, IL, USA) version 20.0. Since patients were not involved, approval by a medical ethics committee was not necessary for this study.

## 3. Results

The response rate to the questionnaires was 100% (*n* = 92): in all hospitals at least one gynecologist completed the questionnaire. A miscarriage protocol was present in all academic hospitals versus 68% of the teaching hospitals and 38% of the nonteaching hospitals (*P* = 0.008 for academic versus nonacademic hospitals).

The median number of women with a miscarriage per hospital per year was 150 (IQR 100–300) in the academic hospitals, 200 (IQR 150–250) in nonacademic teaching hospitals, and 120 (IQR 100–200) in nonteaching hospitals. Annually, approximately 1050 women with an early pregnancy loss presented in the academic hospitals, compared to 6600 women in teaching hospitals and 6550 women in nonteaching hospitals. All academic hospitals offered misoprostol as a treatment option compared to 97.3% of teaching hospitals and 95.7% of nonteaching hospitals. All hospitals offered expectant management and curettage as treatment options to their patients.

According to our respondents, in total, 49% of women underwent curettage as first-choice treatment; 31% of women were treated with misoprostol, while 21% were managed expectantly in the past year. Curettage was first-choice treatment in 29% of patients in academic hospitals versus 46% and 54% of patients in teaching and nonteaching hospitals, respectively (*P* = 0.007 academic versus nonacademic). Misoprostol was first-choice treatment in 41% of patients in academic hospitals versus 34% and 27% of patients in teaching and nonteaching hospitals. There was a significant difference between academic and nonteaching hospitals (*P* = 0.045). Expectant management was first-choice treatment in 30% of patients in academic hospitals versus 20% and 19% of patients in teaching and nonteaching hospitals (Table 1).

For treatment with misoprostol, 23 different regimens were being used. In 15 hospitals, more than one regimen was used. Misoprostol was administered vaginally by 7/8 (87.6%) of academic hospitals, 36/37 (97.3%) of nonacademic teaching hospitals, and 39/47 (83.0%) of nonteaching hospitals. Oral administration was used by 3/8 (37.6%) of academic hospitals, 5/37 (13.5%) of nonacademic teaching hospitals, and 15/47 (31.9%) of nonteaching hospitals. The combination of oral followed by vaginal administration was used by 2/8 (25.0%) of academic hospitals and 9/47 (19.1%) of nonteaching hospitals (Table 2). The initial misoprostol dosages ranged from 200 to 1200 *μ*g.

The preferred method of 51 hospitals was a vaginal dose of 800 *μ*g, which was applied in 63% of the academic hospitals, in 62% of the teaching hospitals, and in 47% of the nonteaching hospitals (*P* = 0.89). In case of no or incomplete expulsion of the miscarriage after the first dose, a repeat dose was administered in all academic hospitals and in 84% and 72% of the teaching and nonteaching hospitals (*P* = 0.32 academic versus nonacademic). A large variation existed in both the frequency and dosage of misoprostol gifts. One academic hospital applied 400 *μ*g of misoprostol twice daily during two days, and one academic hospital applied a once-only repeat dose of 400 *μ*g. The other academic hospitals used repeat doses of 800 *μ*g. Five nonacademic hospitals applied a dosage of 400 *μ*g twice daily, for at least two days. One of these hospitals prescribed 400 *μ*g of misoprostol twice daily for five days. Of nonacademic hospitals, 39/84 (46%) applied repeat dosages of 800 *μ*g once only, three hospitals (3.6%) of 600 *μ*g, and seven hospitals (8.3%) of 400 *μ*g. Other dosages mentioned were once-only repeat dosage of 200 *μ*g (*n* = 4), once-only repeat dosage of 1200 *μ*g (*n* = 1), and 800 *μ*g twice daily during 2 days (*n* = 1). One nonteaching hospital reported that, as part of routine, almost all patients underwent a curettage after their first dosage of 400 *μ*g misoprostol.

The addition of mifepristone to the treatment with misoprostol was applied in 38% of the academic hospitals, 27% of the teaching hospitals, and 45% of the nonteaching hospitals (*P* = 0.47).

In 30% of patients, initial treatment with misoprostol was followed by curettage (academic hospitals median 25%, IQR 25–35; nonacademic teaching hospitals median 30%, IQR 20–50; nonteaching hospitals median 30%, IQR 20–50). The main reasons for performing curettage after initial medical treatment with misoprostol were the sonographic finding of incomplete evacuation of the uterus (84.8%) and/or excessive blood loss (54.3%). In 25% of cases curettages after initial medical treatment were performed on patients' request only, despite the lack of medical reasons (i.e., persisting or excessive blood loss, pain, and/or fever).

In comparison to the survey performed in 2005, the number of clinics prescribing misoprostol for first trimester miscarriage has doubled. Any other comparison could not be made since the 2005 survey focussed on the use of misoprostol for induced abortion.

The most important method for counselling patients about various treatment options was oral information (provided by 100% of the hospitals). In 81.5% of hospitals, gynecologists also referred women to informative websites. In addition, 65.2% of hospitals provided a leaflet with written information.

## 4. Discussion

In this study, we investigated the current practice in miscarriage treatment in Dutch hospitals and especially the implementation of misoprostol treatment. Our main findings were that there is a large practice variation. Despite the availability of noninvasive and cost-effective alternatives, curettage is still the first-choice treatment in 49% of patients. When misoprostol is offered as treatment, the regimens in terms of dosage, frequency of administration, administration routes, and addition of mifepristone vary largely between hospitals. Although academic, teaching, and nonteaching hospitals generally provide care for different types of patients, this is generally not the case where miscarriages are concerned. We do not think it is likely that bias was introduced by patient characteristics.

We inquired about the estimated number of miscarriages per year, the provided treatment options, and the distribution of patients among these management options. Although gynaecologists were asked to provide exact numbers if available, most respondents provided us with estimated numbers instead of exact data. Since the response rate was 100%, we believe that our findings are the second best indicator for current practice in The Netherlands, where a central registration of nonsurgical treatment is not available.

RCOG (NICE) [2] and FIGO [12] have guidelines addressing management of miscarriages and misoprostol use that are applicable to Dutch practice. A possible explanation for the lack of such a guideline in The Netherlands is the divided care for pregnant women. In case of a miscarriage, medical or surgical treatment is always performed by an obstetrician or gynaecologist, but expectant management also takes place in primary care (general practitioner or midwife). In some hospitals, miscarriage treatment is sometimes considered an obstetrical problem, whereas, in other hospitals, it is part of the department of gynecology and/or reproductive medicine. The high response rate to our questionnaire indicates that the subject is considered important by most gynaecologists in The Netherlands. With so many caretakers involved in the treatment of miscarriage, it could be that no one in particular feels the responsibility to initiate a multidisciplinary guideline. Creating a guideline for and with all these caretakers is a difficult task.

Compared to the earlier survey in 2005 [11], the number of hospitals offering misoprostol doubled. This treatment implementation however was not coordinated by the NVOG or another national institute, which results in the use of more than 20 different local protocols regarding medical treatment. The most frequently described misoprostol dosage in literature is a vaginal dose of 800 *μ*g, which can be repeated after 24 to 48 hours in case of nonexpulsion after the first dose [4–9, 13, 14]. The NICE guideline “Ectopic Pregnancy and Miscarriage” by the Royal College of Obstetricians and Gynaecologists (RCOG, 2012) advises giving a single dose of 800 *μ*g misoprostol vaginally or orally according to patients' preference [2].

The International Federation of Gynecology and Obstetrics (FIGO) advises giving 800 *µ*g misoprostol vaginally or 600 *µ*g sublingual, with a repeat dose after 3 hours (maximum of 2 doses) according to a practice bulletin in 2012 [12]. Despite not being distributed actively to Dutch gynecologists, these guidelines and the research on which they are based are easily accessible. However, a distressing number of local protocols in The Netherlands still seem to be based on personal preference or experience since there is no evidence in literature for the efficacy of their misoprostol regimens. When we asked gynecologists for misoprostol regimens used in their hospitals, we found that, in 15 hospitals, more than one regimen was used. We did not ask the gynecologists for the reasons of using different regimens. An explanation for this nonadherence to a local protocol might be personal preference of the patient. For example, if a patient does not want to take vaginal medication, oral medication is prescribed. There might be a lack of knowledge because of insufficient implementation of the local protocol. Furthermore, a negative experience of the individual gynecologist with the dosages mentioned in the local protocol can lead to deviation from this protocol. A dosage of 800 *μ*g misoprostol is the most effective according to literature. Treatment regimens that are suboptimal because the dosage used is higher than 800 *µ*g or lower than 600 *µ*g [2, 12, 14], lead to over- or under treatment of a large group of patients, and deprive these patients from the most efficacious and patient friendly treatment.

A reason why many gynecologists are still reluctant in offering medical treatment might be the risk of incomplete evacuation of the uterus. This occurs in 20–30% of patients treated with misoprostol [4–9, 13]. We did not ask for time duration between misoprostol administration and sonographic follow-up. This might influence the success rate of the treatment. Many cases with sonographic signs of incomplete miscarriage do not evolve into serious clinical problems, such as excessive bleeding, pain, or infection, but are likely to resolve spontaneously. Results of a randomized clinical trial comparing curettage with expectant management in case of incomplete evacuation of the uterus, the MisoREST trial (NL38637.018.11), are awaited in 2015 [15]. If expectant management turns out to be safe and effective, this could potentially save another 3000 surgical procedures (i.e., curettages) in The Netherlands each year. Furthermore, clear evidence on this subject might lead to a further implementation of misoprostol treatment.

Literature on addition of 200 mg oral mifepristone 24 to 48 hours before misoprostol treatment for miscarriage showed conflicting results [14, 16]. To evaluate the efficacy of mifepristone prior to treatment with misoprostol for a nonvital pregnancy, a new trial (M & M trial, NL43938.091.13) will start soon.

There are no differences between the use of misoprostol and curettage with regard to the duration of blood loss, the risk of pelvic infections, and subsequent fertility [9, 10, 13]. Treatment with misoprostol is more cost effective than primary curettage [17, 18]. Despite this, nonacademic hospitals significantly more often applied curettage as first-choice treatment compared to academic hospitals. This might result from infrastructural differences between smaller nonacademic hospitals and academic hospitals which have longer waiting lists. Furthermore, expectant management or misoprostol treatment requires more visits to the outpatients department. Another explanation is that academic hospitals are more aware of international research findings and the implementation of their results and therefore are more willing to adopt new treatment strategies. Also, the insurance system, where the financial compensation differs between surgical and medical treatment, could play a role. This might explain the relatively slow implementation of medical management in daily practice, despite its being a noninvasive and cost-effective option. This is also unfortunate in view of other disadvantages of curettage. A cost-effectiveness analysis in Dutch hospitals showed the costs of first-choice misoprostol treatment to be € 550 lower than the costs of first-choice curettage [17]. We think that this cost difference is applicable to the current situation in other developed countries. According to our results, an estimated number of 10000 women undergo a curettage as first-choice treatment each year. If these women would be treated with misoprostol instead, assuming an effectiveness of 90%, this would prevent 9000 women from undergoing a curettage thereby saving almost 5 million euros per year in The Netherlands only. One can imagine that the potential savings worldwide are considerable.

Besides the costs, there are even more important reasons not to perform curettage as a first-choice treatment. There is no doubt that a curettage is effective, but it has potential major disadvantages: there is a risk of short term complications like uterus perforation or infections and long term complications. 19% of women with a history of curettage develop intrauterine adhesions varying from mild to severe [19]. Furthermore, there is accumulating evidence that women with a history of curettages are at increased risk of preterm birth in subsequent pregnancies [20, 21]. Therefore, we think expectant management or misoprostol treatment should be first choice because of their noninvasive nature, which is in line with the recommendations of the NICE guideline to use expectant management for 7–14 days as first-line treatment, offer medical management if expectant management is not acceptable to the woman, and perform curettage as primary treatment in exceptional cases only [2].

The large practice variation and the difficulties in substituting medical for surgical treatments are not restricted to miscarriage treatment only. For example, implementation of nonsurgical treatment of uterine fibroids was also delayed despite evidence of its effectiveness [22].

The slow uptake of misoprostol treatment for miscarriage is an international issue, which is not restricted to The Netherlands alone. This might be explained by concerns about infection, which, although not supported by data, withhold healthcare providers from using misoprostol, or by differences in reimbursement between medical and surgical treatment. Globally, it is very likely that societal and political forces impact decisions of health care providers on the use of misoprostol. In some countries, antiabortion activists have succeeded in creating the view that misoprostol use may be unlawful, as it is related to use for abortion of pregnancies, which has led to delayed implementation of misoprostol treatment for miscarriages [23].

Apart from access to all treatment options for miscarriage, the preference of the patient is important and obviously should play a decisive role in the final treatment choice. Therefore, the patient needs to be informed about the effectiveness and risks of all treatment options. Negative experiences of caregivers with medical treatment of miscarriage, which might be based on substandard regimens, could influence this counselling. Therefore, all gynaecological caregivers should have knowledge about best treatment regimens, which can be achieved by implementation of an up-to-date guideline. This does not apply to The Netherlands only. The World Health Organization (WHO) has included misoprostol in the* WHO Model List for Essential Medicines* because of its importance in reproductive health and provides recommendations for its use for obstetric and gynecologic indications [24, 25]. As misoprostol is inexpensive, stable at room temperature, and worldwide available, it is also very useful in undeveloped countries [25].

There is little literature on guideline adherence regarding miscarriage treatment. A Scottish survey published in 2006 showed areas of improvement for guideline implementation [26]. Eight years later, despite WHO recommendations, not all countries have implemented a guideline on miscarriage treatment. This leads to practice variation and unnecessary (surgical) treatment, as our study indicates.

## 5. Conclusion

Although the percentage of gynaecologists that are aware of the availability of misoprostol for miscarriage treatment doubled from 50% in 2005 to virtually 100% in 2013, there is still a large practice variation. In the absence of a guideline, a large number of hospitals still use local protocols for misoprostol treatment that are not evidence based. In nonacademic hospitals, curettage is still primary treatment in 50% of patients despite its potential major disadvantages.

The development and implementation of a national guideline or adoption of an international guideline might prevent women from having an unnecessary curettage. Furthermore, it could reduce practice variation and lead to better overall treatment results and patients satisfaction in women treated with misoprostol.

## Figures and Tables

**Table 1:** Mean number of patients (in percentages) per different treatment options.

	Academic (*N* = 8)		Nonacademic, teaching (*N* = 37)		Nonteaching (*N* = 47)	
	%	SD	%	SD	%	SD
Curettage	29	11	46^a^	20	54^a^	20
Misoprostol	41^b^	8,3	34	20	27	17
Expectant management	31^b^	8,5	20	15	20	12

^a^Curettage as primary treatment, academic versus nonacademic teaching hospitals: *P* = 0.028 and academic versus nonacademic nonteaching hospitals: *P* = 0.002 (Mann-Whitney *U* test).

^b^Misoprostol as a primary treatment, academic versus nonteaching hospitals: *P* = 0.045 (Mann-Whitney *U* test).

^c^Expectant management as primary treatment, academic versus nonteaching hospitals: *P* = 0.023 (Mann-Whitney *U* test).

All other comparisons: NS.

**Table 2:** The route of administration and dosage of misoprostol according to local protocols.

	Academic (*N* = 8)		Nonacademic, teaching (*N* = 36)		Nonteaching (*N* = 46)	
	*n*	%	*n*	%	*n*	%
*Vaginal *	*7 *	*87.6 *	*34 *	*91.9 *	*41 *	*89.1 *
200 *µ*g	0	0.0	2	5.6	4	8.7
400 *µ*g	2	25.0	6	16.7	10	21.7
600 *µ*g	0	0.0	3	8.3	3	6.5
800 *µ*g	5	62.5	23	63.9	23	50.0
1200 *µ*g	0	0.0	0	0.0	1	2.2

*Oral *	*3 *	*37.5 *	*3 *	*8.3 *	*13 *	*28.3 *
200 *µ*g	0	0.0	0	0.0	4	8.7
400 *µ*g	1	12.5	2	5.6	3	6.5
600 *µ*g	1	12.5	0	0.0	4	8.7
800 *µ*g	1	12.5	1	2.8	2	4.3

*Oral followed by vaginal *	*0 *	*0.0 *	*0 *	*0.0 *	*4 *	*8.7 *
200 *µ*g + 800 *µ*g	0	0.0	0	0.0	1	2.2
400 *µ*g + 600 *µ*g	0	0.0	0	0.0	1	2.2
600 *µ*g + 600 *µ*g	0	0.0	0	0.0	2	4.3

*Repeat dose given *	*8 *	*100.0 *	*31 *	*83.8 *	*34 *	*72.3 *
